# Inertia-Constrained Reinforcement Learning to Enhance Human Motor Control Modeling

**DOI:** 10.3390/s23052698

**Published:** 2023-03-01

**Authors:** Soroush Korivand, Nader Jalili, Jiaqi Gong

**Affiliations:** 1 The Department of Mechanical Engineering, The University of Alabama, Tuscaloosa, AL 35401, USA; 2 The Department of Computer Science, The University of Alabama, Tuscaloosa, AL 35401, USA

**Keywords:** reinforcement learning, locomotion disorder, IMU sensor, musculoskeletal simulation

## Abstract

Locomotor impairment is a highly prevalent and significant source of disability and significantly impacts the quality of life of a large portion of the population. Despite decades of research on human locomotion, challenges remain in simulating human movement to study the features of musculoskeletal drivers and clinical conditions. Most recent efforts to utilize reinforcement learning (RL) techniques are promising in the simulation of human locomotion and reveal musculoskeletal drives. However, these simulations often fail to mimic natural human locomotion because most reinforcement strategies have yet to consider any reference data regarding human movement. To address these challenges, in this study, we designed a reward function based on the trajectory optimization rewards (TOR) and bio-inspired rewards, which includes the rewards obtained from reference motion data captured by a single Inertial Moment Unit (IMU) sensor. The sensor was equipped on the participants’ pelvis to capture reference motion data. We also adapted the reward function by leveraging previous research on walking simulations for TOR. The experimental results showed that the simulated agents with the modified reward function performed better in mimicking the collected IMU data from participants, which means that the simulated human locomotion was more realistic. As a bio-inspired defined cost, IMU data enhanced the agent’s capacity to converge during the training process. As a result, the models’ convergence was faster than those developed without reference motion data. Consequently, human locomotion can be simulated more quickly and in a broader range of environments, with a better simulation performance.

## 1. Introduction

An accurate model and simulation of human locomotion is highly desirable for various many applications, such as identifying musculoskeletal features, assessing clinical conditions, and preventing aging and locomotor diseases. Although separated human muscles and limbs have been accurately modeled [1], a holistic and reliable simulation of human locomotion is still under development. The most recent research has shown that reinforcement learning techniques are promising for training human locomotion controllers in simulation environments. Consequently, with an accurate simulation of human locomotion motor control, it would be plausible to noninvasively diagnose locomotion disorders and track the rehabilitation process. In some cases, by feeding the neuromechanical-specific regions of the body in a neural network, only specific features of the region of interest are studied (e.g., neuromechanical control model of the prosthetic hand [2]).

Simulating the musculoskeletal system using deep reinforcement learning (RL) has the potential to overcome the limitations of current control models. Advances in deep learning have allowed for the creation of controllers with high-dimensional inputs and outputs for human musculoskeletal models, the outcomes of which could shed light on human motor control, despite the differences between artificial and biological neural networks [3,4]. Our review of the relevant research begins by examining studies related to musculoskeletal simulation and then delves into the reinforcement algorithms that were utilized for simulation purposes.

### 1.1. Musculoskeletal Simulations

**Figure 1:**
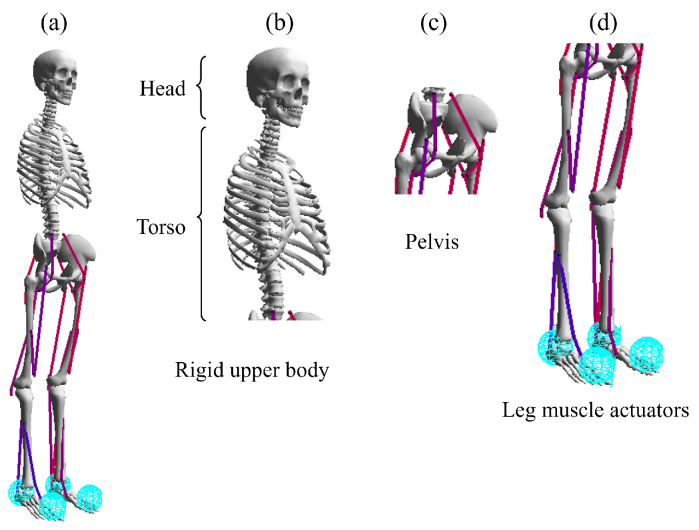
The body sections of the musculoskeletal (**a**), which consist of one upper body segment (**b**), a pelvis (**c**), and several segments for legs (**d**).

**Figure 2:**
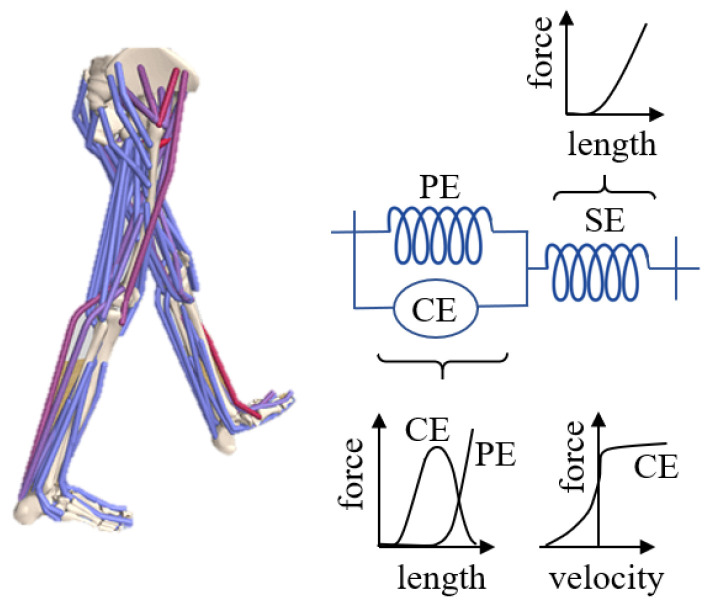
Hill-type muscle models consist of contractile elements (CE), parallel elastic elements (PE), and series elastic elements (SE). Depending on the length and velocity of the contractile element, it produces contractile forces proportional to the excitation signal. Passive elements act as non-linear springs with length-dependent forces.

Musculoskeletal models typically consist of rigid segments and muscle-tendon actuators [5,6,7] (Figure 1) that are connected by rotational joints, which are usually actuated using Hill-type muscle models [8]. These models factor into both active and passive contractile elements [4,7,9,10] (Figure 2) and can be utilized in simulations to estimate metabolic energy consumption and muscle fatigue. The parameters of these models can be adjusted based on an individual’s height, weight, and imaging data such as CT and MRI scans [11,12] and OpenSim [13], and are typically derived from measurements taken from a large sample of people and cadavers [14,15,16]. OpenSim [13] is a widely used open-source biomechanics software package, which serves as the foundation for the OpenSim-RL package [1] used in the Learn to Move competition and is capable of simulating musculoskeletal dynamics [4].

A wide variety of human motion recordings have been analyzed through research efforts in musculoskeletal simulations. By utilizing different types of computational methods, the activation of muscles is identified in a uniform manner, enabling the tracking of reference motion data such as motion capture data and ground reaction forces while reducing muscle exertion [17,18]. The simulation allows for the estimation of body states, such as individual muscle forces, that are challenging to measure directly. This approach has been validated for human walking and running by comparing simulated muscle activation to recorded electromyography data [19,20]. The use of motion-tracking techniques has been demonstrated in the prediction of locomotion diseases [21,22], analysis of human locomotion [17,23], control of assistive devices [24,25,26], and forecasting the impact of exoskeleton assistance and surgical interventions on muscle coordination [27,28]. However, it is noteworthy that, while these simulations can analyze recorded motions, they are unable to predict movement in new scenarios as they do not generate new motions [4].

Through trajectory optimization methods, it is also possible to create musculoskeletal motions without reference motion data [29]. This approach determines the muscles that generate the desired motion by optimizing muscle activation patterns and the musculoskeletal model, with the assumption that the target motion is optimally generated. As a result, this method has produced skilled motor tasks such as walking and running [30,31], as well as insights into the optimal gait for different goals [32,33], biomechanical characteristics [34], and assistive devices [35]. However, when a behavior has yet to be adequately trained and is functionally suboptimal, the application of this method becomes complicated. For example, when wearing lower-leg exoskeletons, people tend to initially walk inefficiently and eventually adapt to more energy-efficient gaits over time [36]; therefore, trajectory optimization based on energy minimization would not accurately predict their initial gait. In addition, physiological control constraints, such as neural transmission delays and limited sensory information, limit human brain function by producing suboptimal behaviors, as the nervous system is optimized for typical motions such as walking. A more accurate representation of the underlying controller may be required to predict the emergent behaviors that deviate from minimum-effort optimal behavior [37].

### 1.2. Reinforcement Learning for Simulation of Human Locomotion

**Figure 3:**
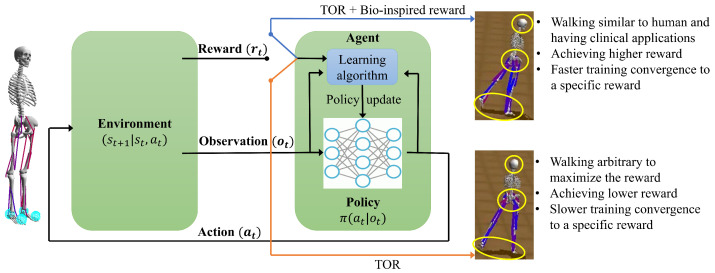
Reinforcement learning algorithm with the reward function consisting of trajectory optimization reward and bio-inspired reward. Employing IMU constraints in the reward function enhances the musculoskeletal simulation with RL by making the locomotion similar to human walking. The circled regions with yellow show the head, pelvis, and legs’ directions during waking. When IMU data is used in training, the gaits are straight and similar to the human. In contrast, when no IMU data is used in training, the agent walks inefficiently, which is not similar to real human walking.

A reinforcement learning paradigm is an approach to solving decision-making problems using machine learning. Hence, through interactions with its environment, an agent tries to optimize its policy π to maximize its cumulative reward [38] (Figure 3). Higher cumulative rewards can be obtained with better-followed target velocities and lower muscle effort in this study’s musculoskeletal model and physics-based simulation environment. A general RL problem involves receiving observations $o_{t}$ at timestep *t* and querying its policy for the action $a_{t}$ (excitation values of the muscles in the model) at timestep *t*. Observations are full or partial descriptions of the state of the environment at timestep *t*. $\pi (a_{t}|o_{t})$ can be either stochastic or deterministic, with a stochastic policy defining a distribution over actions at timestep *t* [39,40,41]. It is possible to calculate gradients from non-differentiable objective functions [42], such as those generated from neuromechanical simulations, and then use the gradients as a basis for updating the policies. After applying the action in the environment, the agent transitions to a new state $s_{t+1}$ and receives a scalar reward $r_{t}\;=\;r\left(s_{t},a_{t},s_{t+1}\right)$. Using a dynamics model, we determine the state transition $\rho (s_{t+1}|s_{t},a_{t})$. A policy should be learned that maximizes the agent’s cumulative reward.

A significant step in solving the RL problem is the proper selection of the policy representation. In this regard, deep RL, achieved by combining the RL with a deep neural network, has been applied as a key solution by modeling the policy that maps the observations to actions in different fields [43,44,45,46]. In the OpenSim-RL environment [1], the actions that drive the musculoskeletal are continuous excitation of the muscles. When the actions are continuous, the model-free deep RL could play a crucial role in developing a model that focuses on maximizing the reward through a direct learning of the policy, rather than the dynamic model of state transition. Hence, defining a proper reward function is a cornerstone to this approach’s success. To this end, the policy gradient algorithm initially approximates the expected reward gradient using the trajectories obtained from policy forward simulation. Then, the policy would be renewed and enhanced based on the reward feedback via gradient ascent [47]. In this way, a more accurate value would be assigned to the continuous actions space (e.g., muscle excitation in OpenSim-RL). Although the standard policy gradient is simple, it has some disadvantages. This approach suffers from instability and inefficiency in sampling. The reason for instability mainly lies in the fact that the gradient estimator may contain high levels of variance; hence, numerous training samples are required for an accurate gradient approximation. New algorithms such as TRPO [48] and PPO [49] have emerged to address the stability issue. These approaches restrict the changes in the policy behavior after each iteration. To this end, the measure of relative entropy is used to control the modifications of policy behavior.

Policy gradient methods are limited by their low sample efficiency. In standard policy gradient algorithms, the gradient is estimated from a new batch of data collected with the current policy at each iteration of policy updating, resulting in each batch of data being used only a few times before being discarded. This often requires millions of sample data to be collected, even for relatively simple problems. Off-policy gradient algorithms, on the other hand, allow for the reuse of data from previous iterations, greatly reducing the number of samples that are required [50,51,52]. An off-policy algorithm, such as DDPG [50], estimates the policy gradient by fitting a *Q*-function, Q(s,a), which represents the expected return after taking a certain action in the current state. The learned *Q*-function is then differentiated to approximate the policy gradient, and the policy is updated using the obtained gradient. SAC and TD3 are recent off-policy methods that offer both improved sample efficiency and stability through various modifications.

The application of deep RL to high-dimensional parameter controllers has also been shown to yield promising results [49,50]. An advantage of deep RL is that it enables the learning of controllers based on low-level, high-dimensional representations of the underlying system, reducing the need to manually design compact control representations and obtaining a deeper understanding of motion. As a result of the development of deep RL models, controllers for complex environments as well as complex musculoskeletal models have been trained [53,54,55]. Moreover, deep RL is compatible with cases where reference motion data can be used to develop the controller [54,56,57]. In this regard, IMU sensor data, as they are inexpensive and easy to collect in various environments (except when exposed to an environment with extensive varying magnetic fields), are very desirable for employment as the reference motions [58].

## 2. Materials and Methods

Our approach integrates the use of Soft Actor-Critic (SAC) and Recurrent Experience Replay within the framework of Distributed Reinforcement Learning to handle both continuous and discrete action spaces. We adopt a hybrid training strategy that combines bio-inspired rewards and TOR (Figure 3).

We used the L2M2019 environment of OpenSim-RL [1] for our simulation. This environment provides a physiologically plausible 3D human model to move following velocity commands with minimal effort [1]. This human model (Figure 1a) consists of a single segment showing the upper section of the body (Figure 1b), a pelvis segment (Figure 1c), and several segments for legs (Figure 1d).

In the environment, the head and torso are considered a single rigid segment and connected to the pelvis via a ball-and-socket joint. The orientation of the torso with respect to the pelvis is specified through ZXY rotations, which denote lumbar extension, bending, and rotation, respectively. The primary function of the upper body in this environment is to follow the overall movement of the torso and upper limbs during walking, rather than replicating intricate upper body kinematics such as spinal bending or intricate scapular movements [6].

The leg muscles used in this environment are broken down into 22 different muscles, with 11 for each leg. These muscles include the hip abductor and adductor; hip flexor and extensor (glutei); hamstrings, which are a combination of hip extensor and knee flexor; rectus femoris, which acts as both a hip flexor and knee extensor; vastii, which acts as a knee extensor; biceps femoris short head, which acts as a knee flexor; gastrocnemius, which acts as both a knee flexor and ankle extensor; soleus, which acts as an ankle extensor; and tibialis anterior, which acts as an ankle flexor. Additionally, the environment uses eight internal degrees of freedom (four for each leg), which are used to control the hip abduction/adduction, hip extension/flexion, knee extension/flexion, and ankle plantar flexion/extension movements.

### 2.1. Simulation Environment

**Figure 4:**
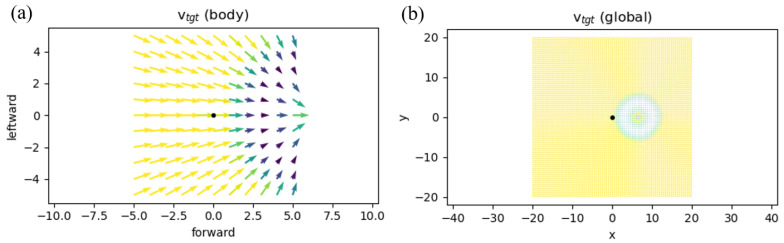
The body target velocity guiding the musculoskeletal body toward the coordinates (5,0) is shown in (**a**). The global environment in which the musculoskeletal should reach the coordination of (5,0) is illustrated in (**b**).

The locomotion controller receives its inputs from (1) a local target velocity *V* and (2) the musculoskeletal state *S*. The components of the states are pelvis state, muscle states, ground reaction forces, joint angles and rates. These states provide 97 values for the observable states [1]. In addition, as shown in Figure 4a, a 2D vector field on an 11 × 11 grid in the environment provides a 2 × 11 × 11 matrix, showing a local target velocity field. In this figure, each square block has 0.5 m in length and width. Each of the 0.5 × 0.5 blocks has a 2D vector demonstrating the target velocities. The agent starts at the coordination of [0,0] and the target coordination is [5,0] (Figure 4b). The action space consists of a vector, with 22 values showing the activation of 22 muscles (11 per leg).

**Figure 5:**
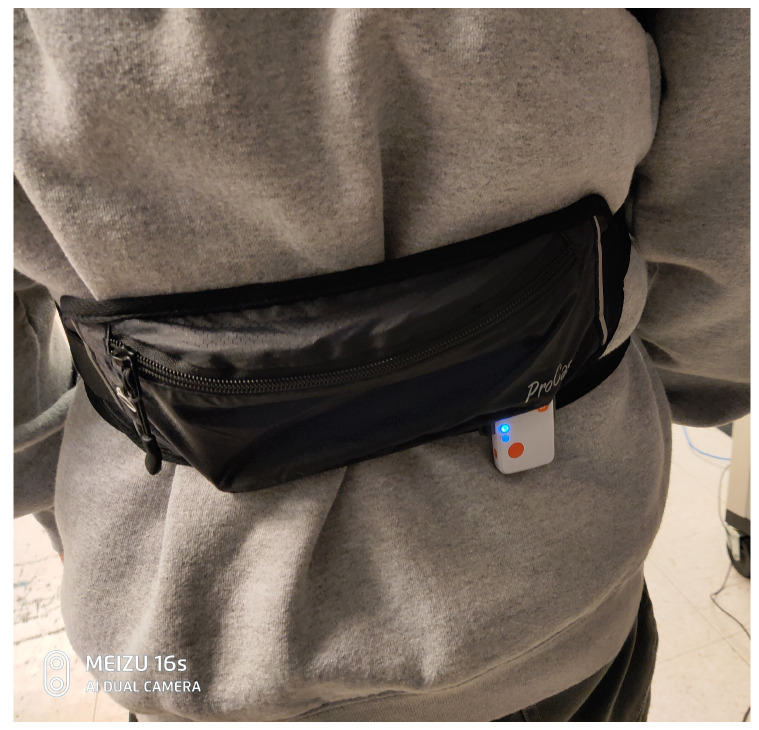
The attached IMU to the participant’s shimmer for data collection during straight walking.

Moreover, the environment offers different difficulty levels in this environment. Although at difficulty = 2 the environment assigns a random location as the target for the agent; at difficulty = 1, the target is located at the coordination of [5,0] (Figure 4). This scenario is the same as in our data collection process. In our data collection process, one 35-year-old male participant with 170 cm height and 75 Kg weight walked in a straight line for 5 m at a comfortable speed. The reason for selecting this task is that the 5-meter walk test task has shown promising results in the detection of the diseases such as Parkinson’s disease [59]. In addition, the Society of Thoracic Surgeons recommends this task for the Adult Cardiac Surgery Database as an effective measure to predict frailty among candidates for cardiac surgery [60]. Moreover, an already-developed RL simulation environment drove the researcher to select the 5 m walking task. The short duration of the walking task means that it avoids the substantial data size generation in simulation, and the data collection process in the experiment makes this task an appealing subject of investigation using the RL approach. These advantages have made the 5 m walk an attractive task for locomotion assessments [61]. As shown in Figure 5, the IMU sensor, Shimmer, was connected to the pelvis of the participant. Then, a penalty was considered for deviations from the observed IMU values of the agent in the environment derived from the collected IMU values. This constraint was used to build the bio-inspired reward of our algorithm. For TOR, we used the defined reward at [55]. The following equations show the defined reward functions:

The total reward *J*(π) is high when the human model locomotes at desired velocities with minimum effort:

(1) $$\begin{array}{c} J\left(\pi \right)\;=\;R_{alive}+R_{step}\;=\;\sum_{i}r_{alive}+\sum_{i}r_{step}\left(w_{step}\cdot r_{step}-w_{vel}\cdot c_{vel}-w_{eff}\cdot c_{eff}\right) \end{array}$$

where *R_alive_* prevents the agent from falling and the step term urges the agent to move toward the target, which, here, is shown by a coordination of [5,0] on Figure 4a. In the OpenSim-RL [1], *r_alive_*, *r_step_*, *c_vel_*, and *c_eff_* are defined as:

(2) $$\begin{array}{cc} \; & r_{alive}\;=\;0.1\\ \; & r_{step}\;=\;\sum_{i\;in\;\;step_{i}}\Delta t_{i}\;=\;\Delta t_{step_{i}}\\ \; & c_{vel}\;=\;||\sum_{i\;in\;\;step_{i}}\left(v_{vel}-v_{tgt}\right)\Delta t_{i}\left|\right|\\ \; & c_{eff}\;=\;\sum_{i\;in\;\;step_{i}}\sum_{m}^{muscles}A_{m}^{2}\Delta t_{i} \end{array}$$

in Equation (2), Δ = 0.01 s is the simulation timestep, $v_{vel}$ is the velocity of the pelvis, $v_{tgt}$ is the target velocity, $A_{m}$s are the muscle activation, and $w_{step}$, $w_{vel}$, and $w_{eff}$ are the weights for the stepping reward, velocity and effort.

The objective of this paper is to simulate a musculoskeletal agent walking similarly to the participant, where IMU data have been collected using reinforcement learning. To this end, the RL is utilized to develop a policy π(*a_t_*|*s_t_*) that can maximize the discounted sum of the expected rewards:

(3) $$\begin{array}{cc} \; & J\left(\pi \right)\;=\;\sum_{t}E_{\left(s_{t},a_{t}\right)∼\rho_{\pi }}\left[\gamma^{t}r\left(s_{t},a_{t}\right)\right] \end{array}$$

where *s*_t_ ∈ S is state, *a*_*t*_∈ A is action, *r*: S × A → [*r*_min_, *r*_max_] is reward function and $\rho_{\pi }$ represents the state-action marginals of the trajectory distribution induced by the policy π(*a_t_*|*s_t_*). As the main RL procedure, as described in [55], the Soft Actor-Critic algorithm [52,62] was used. SAC is the latest and most advanced version of DDPG, which is a type of machine learning algorithm for situations in which the available actions are continuous. DDPG is considered efficient, as it allows for the reuse of previous data to update the current policy. SAC works by balancing two objectives, maximizing reward and maximizing entropy, to achieve stable and efficient learning. SAC has been shown to be highly effective, with good data efficiency, stability in learning, and robustness to changes in its parameters. This is accomplished by the addition of a new entropy term to the reward Equation (3).

(4) $$\begin{array}{cc} \; & J\left(\pi \right)\;=\;\sum_{t}E_{\left(s_{t},a_{t}\right)∼\rho_{\pi }}\left[\gamma^{t}\left(r\left(s_{t},a_{t}\right)+\alpha H\left(\pi \left(\times |s_{t}\right)\right)\right)\right] \end{array}$$

where α is a trade-off between the entropy and reward and thus controls the stochasticity of the optimal policy. Reinforcement learning methods using off-policy continuous action spaces are based on the actor–critic pair, where the critic estimates *Q*-value:

(5) $$\begin{array}{c} Q_{\pi }\left(s_{t},a_{t}\right)\;=\;r\left(s_{t},a_{t}\right)+\sum_{k=t+1}E_{\left(s_{k},a_{k}\right)∼\rho_{\pi }}\left[\gamma^{k}\left(r\left(s_{k},a_{k}\right)+\alpha H\left(\pi \left(\times |s_{t}\right)\right)\right)\right] \end{array}$$

In practice, actor and critic are represented by neural networks $\pi_{\phi }$ (*a_t_*|*s_t_*) and $Q_{\theta }$ (*s_t_*|*a_t_*) with parameters ϕ and θ. Standard practice is to estimate the mean and variance of factorized Gaussian distribution, $\pi_{\phi }\left(a_{t}|s_{t}\right)\;=\;N\left(\mu_{\phi }\left(s_{t}\right),\sum_{\phi }\left(s_{t}\right)\right)$. A distribution such as this allows for reparametrization and policy training through backpropagation. Using this parametrization, the learning objectives for actor, critic, and entropy parameters read as follows:

(6) $$\begin{array}{cc} \; & J_{\pi }\left(\phi \right)\;=\;E_{s_{t}∼D}\left[E_{a_{t}∼\pi_{\phi }}\left[\alpha \log\left(\pi_{\phi }\left(a_{t}|s_{t}\right)\right)-Q_{\theta }\left(s_{t},a_{t}\right)\right]\right],\\ \; & J_{Q}\left(\theta \right)\;=\;E_{(s_{t},a_{t})∼D}\left[\frac{1}{2}{\left(Q_{\theta }\left(s_{t},a_{t}\right)-\left(r\left(s_{t},a_{t}\right)+\gamma E_{s_{t+1}∼p}\left[V_{\bar{\theta }}\left(s_{t+1}\right)\right]\right)\right)}^{2}\right],\\ \; & J\left(\alpha \right)=E_{a_{t}∼\pi_{t}}\left[-\alpha \log\pi_{t}\left(a_{t}|s_{t}\right)-\alpha \bar{H}\right] \end{array}$$

Experience replay (ER), denoted by *D*, and the objectives can be optimized through the use of various methods for stochastic gradient descent. In addition to the previously mentioned benefits, the policy also has the advantage of continuously exploring promising actions and discarding those that are not effective.

In reinforcement learning, ER is a commonly used data storage technique for off-policy algorithms. As an agent interacts with its environment, it records transactions consisting of the state (*s*), action (*a*), reward (*r*), and next state ($s^{\prime }$) it receives. A variation of this method, called Prioritized Experience Replay [63], prioritizes transactions based on the amount of associated loss, ensuring that higher-loss transactions are more likely to be used during training. In the R2D2 approach, the transaction itself is not stored in ER, but overlapping sequences of consecutive (s,a,r) transactions. Sequences never cross episode boundaries and overlap by half-time steps. These sequences are referred to as segments. The R2D2 pipeline uses n-step prioritization to determine the priority of each segment. This method is based on the n-step TD-errors $\delta_{i}$ over the sequence: $p\;=\;\eta \;max_{i}\;\delta_{i}+\left(1-\eta \right)\;\bar{\delta }$, where η is set to 0.9.

### 2.2. Reward Shaping

The reward function is pivotal to RL agents’ behavior: they are motivated to maximize the returns from the reward function, so the optimal policy is determined by the reward function [55]. Sparse and/or delayed rewards can make learning difficult in many real-world application domains. RL agents are typically guided by reward signals when interacting with their environment. Learning speed and converged performance can be improved by the addition of a shaping reward to the reward that is naturally received from the environment, which is called the reward shaping principle. Nonetheless, there are two main problems in using reward shaping in RL [55]: (1) Interference of rewards—for example, moving with minimum effort is desired; however, to define the reward function, a velocity bonus can be used to sum up with an effort penalty. (2) Difficulty modifying the existing rewards—when the agent learned, through a reward, to take an action, but the action cannot fully achieve a specific purpose, e.g., moving a leg but not moving forward. Hence, modifications to the reward function are needed, which can cause the previously learned action to be forgoteen.

To address these two issues, a *Q*-function split technique called multivariate reward representation is introduced [55], in which the scalar reward function is weighted as the sum of the *n* terms:

(7) $$\begin{array}{c} r_{t}\;=\;\sum_{i=1}^{n}w_{i}\times r_{i,t} \end{array}$$

In this approach, the reward terms do not interfere with each other as each term is used separately and the corresponding *Q*-function of each reward term is optimized. Accordingly, if more physiological rewards are collected, more reward functions based on realistic human locomotion can be added to this reward function, which makes this algorithm a suitable choice for a combination of TOR and bio-inspired physiological data. This multivariate reward approach allows for the critic pretraining to add new reward terms or remove the existing reward terms. The critic is represented by the neural network. To remove a reward, the parameters assigned to the reward can be set to zero; to add a new reward, the matrix should be extended by the addition of a new row. To train the actor and critic with multivariate reward representation, the vector of critic loss should be optimized, and the actor should optimize its policy with the scalar representation of the *Q*-function:

(8) $$\begin{array}{cc} \; & Q\left(s_{t},a_{t}\right)\;=\;\sum_{i=1}^{n}w_{i}\times Q_{i}\left(s_{t},a_{t}\right) \end{array}$$

The reward function used here is:

(9) $$\begin{array}{c} \bar{r}=\left[r_{env},r_{clp},r_{vdp},r_{pvb},r_{dep},r_{tab},r_{entropy},r_{IMU}^{roll},r_{IMU}^{pitch},r_{IMU}^{yaw}\right] \end{array}$$

To evaluate the addition of the bio-inspired rewards, we kept the reward function used in [55]; however, three terms, $r_{IMU}^{roll},r_{IMU}^{pitch},r_{IMU}^{yaw}$, were added to the reward function and, thanks to the multivariate reward representation, do not interfere with the other rewards in the training process. $r_{IMU}^{roll},r_{IMU}^{pitch},r_{IMU}^{yaw}$ are defined as the deviation of the collected IMU data $IMU_{col}$ from the IMU data $IMU_{obs}$ observed in the environment during training.

(10) $$\begin{array}{cc} \; & r_{IMU}^{roll}=-\left|IMU_{col}^{roll}-IMU_{obs}^{roll}\right|\\ \; & r_{IMU}^{pitch}=-\left|IMU_{col}^{pitch}-IMU_{obs}^{pitch}\right|\\ \; & r_{IMU}^{yaw}=-\left|IMU_{col}^{yaw}-IMU_{obs}^{yaw}\right| \end{array}$$

A weight of 1 was assigned to these rewards (Equation (7)). Another benefit is that if more physiological data (e.g., more IMU data from other parts of the body) are collected, the reward function can be extended and more bio-inspired constraints can be added to the reward function. Consequently, the musculoskeletal mimicking the human locomotion tasks can move closer to the real scenarios. The other rewards are defined as follows:

Crossing legs penalty ($r_{clp}$) is defined to stop the agent’s tendency to cross its legs.

(11) $$\begin{array}{c} r_{clp}=min\left(0,\left(r^{head}-r^{pelvis},r^{left}-r^{pelvis},r^{right}-r^{pelvis}\right)\right) \end{array}$$

where *r* is a radius vector. To encourage the agent to move at the early stages, $r_{pvb}$, the pelvis velocity bonus is used:

(12) $$\begin{array}{c} r_{pvb}=\left|\right|v_{body}\left|\right| \end{array}$$

Velocity deviation penalty $r_{vdp}$ is defined to guide the agent toward the target.

(13) $$\begin{array}{cc} \; & r_{vdp}=-\sum_{i\;in\;step_{i}}\left|\right|v_{body}-v_{tgt}\left|\right| \end{array}$$

$r_{dep}$, dense effort penalty, aims to move the agent with minimal effort

(14) $$\begin{array}{cc} \; & r_{dep}=-||action_{t}\left|\right| \end{array}$$

To force the agent to stop at the target, the reward of the target achievement bonus is added $\left(r_{tab}\right)$:

(15) $$\begin{array}{c} r_{tab}=\left\{\begin{array}{cc} 0, & 0.7<||v_{tgt}\left|\right|\\ 0.1, & 0.5<||v_{tgt}\left|\right|\leq 0.7\\ 1-3.5||v_{tgt}{\left|\right|}^{2}, & \left|\right|v_{tgt}\left|\right|\leq 0.5 \end{array}\right. \end{array}$$

The last reward coordinate is the entropy bonus from SAC:

(16) $$\begin{array}{c} r_{entropy}\;=\;\alpha \times H\left(\pi \left(\times |s_{t}\right)\right) \end{array}$$

Finally, in our method, the described multivariate reward function, which is a combination of bio-inspired inertial-constrained and TOR, loss functions and networks from SAC, parallel data collection and prioritization, n-step Q-learning and invertible value function rescaling from R2D2 were used to train the agent.

## 3. Results

To provide a fair judgment when distinguishing the IMU reward’s contribution to developing the agent’s locomotion motor modeling, the training neural network structures should be similar to cases in which no IMU reward was used for training. Hence, similar to [55], to train the RL agent, the musculoskeletal walked in a straight line for 5 m, similar to the human from which the data were collected. We taught the agent to walk in any direction, and then the agent walked in a straight line for five meters. The neural network structure that forms the first step for both critic networks and policy has four hidden layers. The observation (input) size for policy was 97, and for the critic, the size was 119 (22 action values plus 97 states). The hidden layer of the critic has 256 layers. In the case of the activation layer, ‘ELU’ and ‘ReLU’ were employed for policy and critic, respectively. The value of 0.99 was considered as the discount factor, γ. The size of the experience replay was 250,000 and the segment length was ten when using a 30 data sampler. The learning rate of $3\times 10^{-5}$ for policy and $10^{-4}$ for critic was regarded for the Adam optimizer. The batch size of 256 and segment length of ten were used. Priority exponents α and β were set to 0.1 at the beginning of training and linearly increased to 0.9 in 3000 training steps.

The second step of learning, after starting to walk in any direction, is to walk forward, toward the target. To this end, a new model with $\pi_{\phi }^{s}\left(a_{t}|s_{t},v_{t}\right)$ and $Q_{\theta }^{s}\left(s_{t},v_{t},a_{t}\right)$ was trained by minimizing the Kullback–Leibler divergence between policies and mean squared error between critics on data from a previously saved experience replay:

(17) $$\begin{array}{cc} \; & J\pi^{s}\left(\phi \right)=E_{st∼D}E_{v_{t}∼N\left(0,0.1\right)}[D_{KL}(\pi_{\phi }^{s}\left(a_{t}|s_{t},v_{t}\right)\left|\right|\pi_{\phi^{\prime }}^{t}\left(a_{t}|s_{t}\right))]\\ \; & J_{Q^{s}}\left(\theta \right)=E_{st∼D}E_{v_{t}∼N\left(0,0.1\right)}{\left(Q_{\theta }^{s}\left(s_{t},v_{t},a∼\pi^{s}\left(\times |s_{t},v_{t}\right)\right)-Q_{\theta^{\prime }}^{t}\left(s_{t},a∼\pi^{t}\left(\times |s_{t}\right)\right)\right)}^{2} \end{array}$$

Models $\pi^{s}\left(a_{t}|s_{t},v_{t}\right)$ and $Q^{s}\left(s_{t},v_{t},a_{t}\right)$ share the same architecture as $\pi^{t}\left(a_{t},s_{t}\right)$ and $Q^{t}\left(a_{t},s_{t}\right)$, although the input dim is now dim(S) + dim(V) = 97 + 2 × 11 × 11 = 339 for policy and dim(S) + dim(V) + dim(A) = 339 + 22 = 361 for critic. The hidden size equals 1024 for both. The Adam optimizer [64] with the learning rate $10^{-4}$ was used to optimize the distillation losses for policy and critic networks and batch size 128.

**Figure 6:**
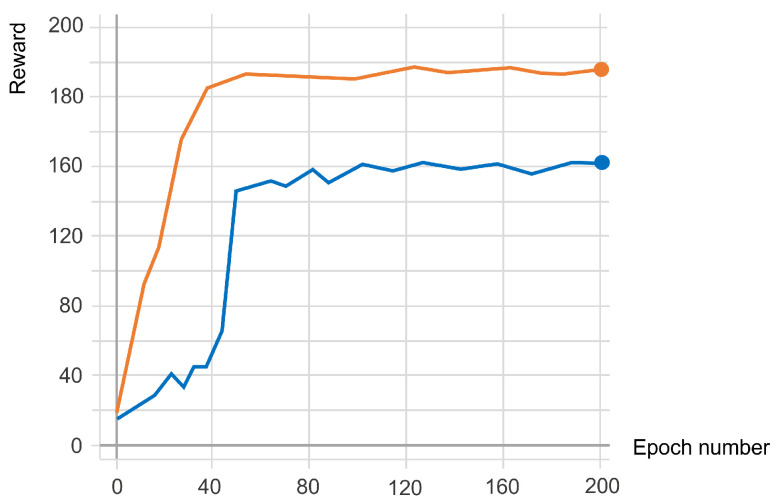
Comparison of the reward obtained by the agent with the same training configuration, when no IMU sensor is used (blue), using the IMU constraints (orange). The horizontal axis shows the training epoch number and the vertical axis shows the reward.

**Figure 7:**
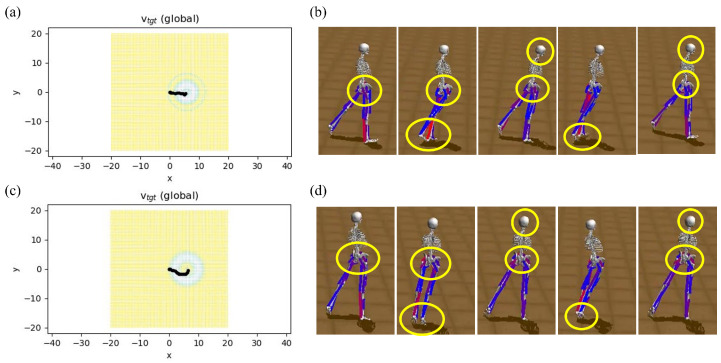
(**a**) The musculoskeletal locomotion trajectory and (**b**) frames for a five-meter straight walk when IMU constraint is used for training. (**c**) The musculoskeletal locomotion trajectory and (**d**) frames for a five-meter straight walk when no IMU constraint is used for training. The highlighted regions in (**b**,**d**) illustrate that when IMU data are used to train the agent, the body direction is straight. Conversely, when no IMU data are used, the agent walks in an inefficient manner and the direction is not straight, unlike a normal human.

**Figure 8:**
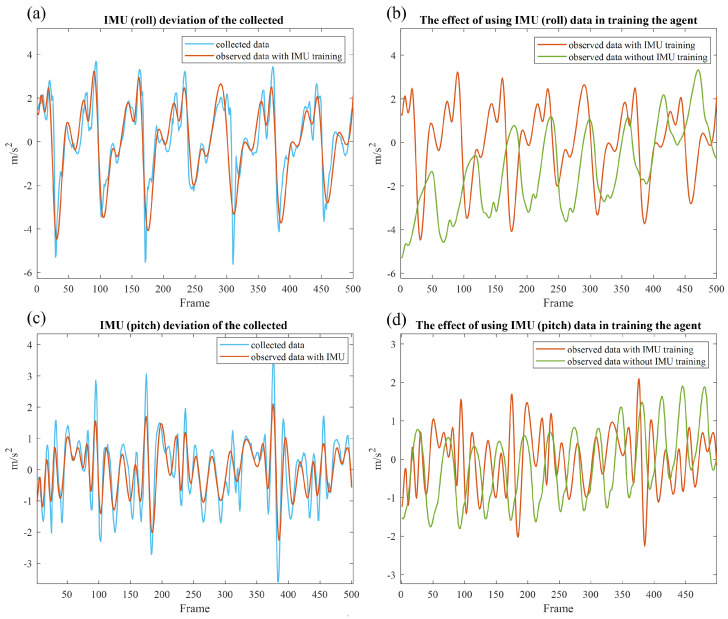

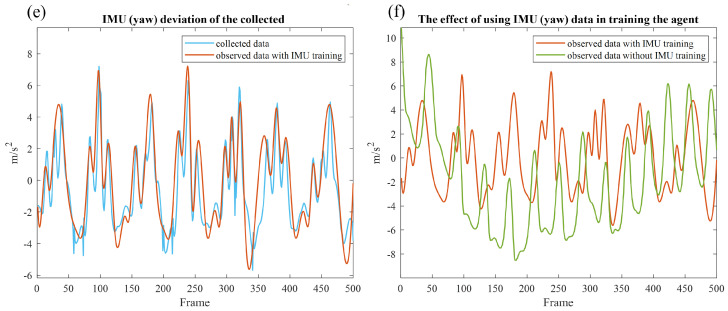
The IMU deviation of the collected IMU data from the observed IMU data (m/s^2^) for the first 5 s of the simulation is shown on the vertical axes, and the horizontal axes show the 500 frames of the timesteps (0.01 s): (**a**) roll, RMSE = 0.8824 (**c**) pitch, RMSE = 0.5825 (**e**) yaw, RMSE = 1.5908. Roll (**b**), pitch (**d**), and yaw (**f**) directions are used to demonstrate the difference in walking directions between an agent trained with IMU data (orange) and an agent trained without IMU data (green).

The explained hyperparameters and steps were taken for both cases, in which no IMU-constrained reward was used and the IMU-constrained reward was used, and the results are shown in Figure 6. In addition to the faster training and higher reward, which were calculated according to the deviation of IMU data from the environmental observations, data recorded from the participant approached zero. In this regard, Figure 7a shows the path that musculoskeletal walks to reach the target spot, which is 5 m from the start point when IMU data are used to train the agent. Compared to Figure 7c, where no IMU constraint was used to guide the agent to the target, the latter case, Figure 7c, shows some deviations from the straight path; however, in Figure 7a the agent walks in a more straightforward way. This is because IMU constraints provide an accurate guideline for the agent to achieve its goal with minimal effort. For further demonstration, the musculoskeletal walking frames when the IMU constraint is used (Figure 7b) and when no IMU constraint is used (Figure 7d) demonstrate the agent’s deviation from walking in a straught line by showing its effect on the manner in which agent takes its steps and adjusts its body direction when no IMU constraint is used. To clarify, the agent’s head, pelvis, and feet are highlighted in these two figures showing that, when IMU reward is used, the agent walks in a straight line, similar to the human from which the data were collected, but it walks in an inefficient way when no IMU data are used. To investigate the agent’s deviation from the locomotion behavior of the participant, the Root Mean Square Error (RMSE) was calculated and the RMSE for roll, pitch, and yaw data was 0.8824, 0.5825, and 1.5908; respectively (Figure 8a,c,e). Moreover, Figure 8b,d,f compares the observed IMU data in the simulation environment when IMU data were used for training (orange) and when no IMU data were used for training (green). There is an increasing trend in the IMU data observed from the simulation environment when no IMU data were used for training.

## 4. Discussion

This study developed an integrative framework for designing a novel reward function of both TOR and bio-inspiration, to develop RL techniques that could model human motor control. The experimental results demonstrate that the models can reduce training time and increase rewards when simulating human locomotion, compared with previous work that did not consider human motion data. Our contributions are: (1) to introduce the novel reward function combining TOR and a bio-inspired reward function, and (2) to demonstrate a computational framework to redesign a reward function and improve human locomotion simulation models.

As shown in Figure 6, the RL model with an IMU-constrained reward function showed a faster learning rate and could achieve a higher reward. Using this approach, the IMU constraints provide a reference guideline, allowing for the agent to walk similarly to natural human movements. These rewards could help the agent achieve a higher reward and faster learning than the model when no bio-inspiration is used. Notably, this finding suggests that integrating real-world data into the reward function of the RL techniques could help the simulation models escape some anomalies or saddle points during the training process. This observation is consistent with other theoretical analyses of deep neural networks’ convergence processes [65].

According to the results shown in Figure 8, the participants’ pelvis motion while walking in a straight line can be replicated through an acceptable training process for RL model training. Hence, with accurate models of human anatomy (e.g., OpenSim [66]), and without any invasive procedure, the participants’ locomotion disorders can be investigated, or at least the pelvis part of the participant can be accurately analyzed. This function will be beneficial for medical applications. For instance, a rehabilitation therapist could import their patients’ IMU sensor data to the RL models. The models could provide estimates of the patients’ musculoskeletal mechanisms to assist the therapist in identifying potential issues during rehabilitation and determining better strategies.

Another significant implication of our research is that the experimental results only rely on a single IMU sensor, used on the participants’ pelvis. In the last decade, despite the advances in wearable and mobile techniques, the affordability and acceptance of sensing techniques constrained existing studies on human locomotion. Using much of the bio-sensor data is challenging, as they are costly to collect. Not all the sensors can be used in all environments, for example, high-resolution cameras in an open environment or electromyography (EMG) data in a laboratory environment. In addition, by increasing the need for remote health monitoring and reducing the need for patients to attend doctors’ clinics, it is very desirable that patients use some easy-to-wear sensors such as IMU sensors and send the data to their doctors. Previous attempts to collect data on human locomotion for healthcare research mainly involved instructing participants to wear a wearable IMU sensor, such as Lumo Run [67]. Lumo Run was originally created to track the pelvic motion of runners for gait analysis and feedback, but it has since been adopted for use in a range of health research studies [68].

With the robust framework developed in this research, their locomotion could be simulated and assessed remotely and retrospectively. The aim of this research is to equip researchers with a simulation model to reinvestigate the existing human locomotion dataset.

## 5. Limitations and Future Work

The limitations of this study are the relatively small sample size, the low number of musculoskeletal tasks in the experiment, the inaccuracy of the computational models, and other factors that occurred during data collection and preprocessing. Because the RL framework is designed for personalized human locomotion modeling, the experiments focused on training, testing, and validating individual participants’ data. Further work will explore the characteristics of the models across participants and quantify the uncertainties that occur during the generalization process. In addition, interpreting the RL results and training process is still challenging, making it difficult to ensure clinical meaningness.

Another limitation to the use of RL is the simulation environment and computational resources. In this respect, besides 5-m walking and clinical gait analysis, a variety of tests can assess a person’s walking pattern, including timed up-and-go tests, a 6 min walk test, treadmill gait analysis, and walk back and forth tests. However, for tasks such as treadmill gait analysis, an environment for RL simulation has not been developed. Some other tasks, such as six-minute walking, require enormous amounts of data generation and collection, which limits the selection of these tasks to the computational reinforcement learning approach.

In future work, we will apply the RL models with more than one IMU constraint to different parts of the body to replicate more complicated locomotion tasks such as jogging, jumping, and running to embrace the knowledge learned from this study. The reason for continuing the research direction of different locomotion tasks is that each of these tasks activates a set of joints and muscle synergy, which move in distinct directions. Hence, for a comprehensive investigation of locomotion disorders, more than one locomotion task simulation is required to investigate the activation of joints and muscles in different locomotion directions (i.e., sagittal, coronal, and transverse).

## 6. Conclusions

Our study showed that the integration of IMU data into the RL framework reward functions could improve human locomotion simulation. In our experiments, IMU data collected from a participant walking in a straightforward way for 5 m were used to train a musculoskeletal model in a simulation environment. Consequently, this bio-inspired constraint could help the agent move its pelvis like the human from which the IMU data were collected. This concept was shown through a comparison of the trajectory (Figure 7a,c), walking frames (Figure 7b,d), obtained RL rewards, and a comparison of the collected IMU data with the IMU data observed in the simulation environment. In this regard, the maximum reward obtained when the IMU constraint was included in the reward function was 190, while, when no IMU constraint was used, the maximum reward was 160 (Figure 6). The RMSEs between the collected IMU reward and the observed reward from the agent in the roll, pitch, and yaw were 0.8824, 0.5825, and 1.5908, respectively (Figure 8). A comparison was made between musculoskeletal agents trained with IMU data and those trained without IMU data. The results demonstrated the improved performance of musculoskeletal agents trained with IMU data, including faster convergence, higher reward, and better simulated human locomotion. These findings are consistent with the existing theoretical work on escaping the saddle points of deep neural networks. Furthermore, we discussed the implications and potential medical applications of these findings.

## Data Availability

Not applicable.
